# Equivalent Calibration Method Based on a Blackbody Baffle Substitution for a Large External Surface-Source Blackbody

**DOI:** 10.3390/s22155844

**Published:** 2022-08-04

**Authors:** Xinyu Pang, Yi Yu, Zhou Li, Zhiyuan Sun, Chun Li, Guoqing Yang

**Affiliations:** 1 Changchun Institute of Optics, Fine Mechanics and Physics, Chinese Academy of Sciences, Changchun 130033, China; 2 University of Chinese Academy of Sciences, Beijing 100049, China; 3 Key Laboratory of Space-Based Dynamic & Rapid Optical Imaging Technology, Chinese Academy of Sciences, Changchun 130033, China

**Keywords:** infrared systems, radiometric calibration, blackbody baffle, equivalent calibration conversion function

## Abstract

Highly accurate measurements of infrared systems cannot be achieved without precise radiometric calibrations. In order to correctly interpret and process infrared images and monitor the performance of infrared cameras, their radiometric calibration is also required periodically. In this paper, an equivalent calibration method is proposed based on an internal blackbody baffle. It is used for the replacement of a large surface-source blackbody covering the aperture for the field calibration of large-aperture equipment. Subsequently, the expressions of the equivalent calibration conversion function (ECCF) are derived based on the grayscale response of the camera and the calibration models of the two methods, and experimental measurements and fits are performed using a cooled mid-wave infrared camera. The results show that the measured functional form is consistent with the physical meaning. Moreover, in the target imaging experiments, the results of the inversion using the equivalent calibration conversion function and the results of the direct calibration of the external blackbody are largely consistent with the average error of 0.198% between the two, and the maximum error is within 1%. The maximum error between the inversion result of radiation brightness and the actual value of the target is 6.29%, and the accuracy fully meets the radiometric measurement requirements.

## 1. Introduction

With the continuous development of infrared imaging and infrared detection technologies, the measurement of infrared radiometric characteristics has become an important tool for feature acquisition and the identification of complex or weak targets [1,2,3,4]. Obtaining quantitative data from an infrared camera requires radiometric calibration, which relates the grayscale values of the digital image output from the camera to physical quantities such as radiance, and establishes a quantitative relationship between the input and output quantities [5,6].

Infrared measurement equipment requires recalibration of the system for calibration data updates before performing measurement tasks in the field. For large-aperture radiometric measurement equipment, it is necessary to prepare large surface-source blackbodies capable of covering that aperture [7,8]. The development cost of large surface-source blackbodies is high, the equipment development is tedious, and the temperature stabilization time is long, making the calibration working time long, the workload heavy, and the equipment maintenance cost high [9,10,11].

This paper presents an equivalent calibration method based on an internal blackbody baffle. After the infrared measurement equipment is calibrated in the laboratory to determine the equivalent calibration conversion function, a simple calibration process using the blackbody baffle can be equivalently converted to an external blackbody calibration covering the pupil when the calibration work is performed in the external field, without the need for a large surface-source blackbody matching the aperture of the equipment, leading to improved calibration efficiency and reduced associated costs.

In Section 2, the response model of the infrared camera detector and the calibration model of the system are analyzed, along with the effect of the ambient temperature on the calibration model. Section 3 provides a detailed theoretical description of the proposed equivalent calibration method, derives the mathematical expression of the equivalent calibration conversion function, and introduces the specific measurement method. In Section 4, calibration experiments and comparative analysis of the data are carried out to verify the above theory. In Section 5, it is concluded that the proposed method is effective and can be used as an alternative to the external large-field surface-source blackbody calibration method, and that the calibration accuracy is no less than that with the direct use of external blackbodies.

## 2. Detector Response of the Infrared Radiation Measurement System

### 2.1. Linear Response of the FPA of the Infrared Camera Detector

Since cooled infrared focal plane arrays are generally better than uncooled focal plane arrays in terms of sensitivity, minimum detection temperature, and other performance metrics [12,13], they are usually suitable for military fields with high requirements for imaging quality and precision measurement.

**Figure 1:**
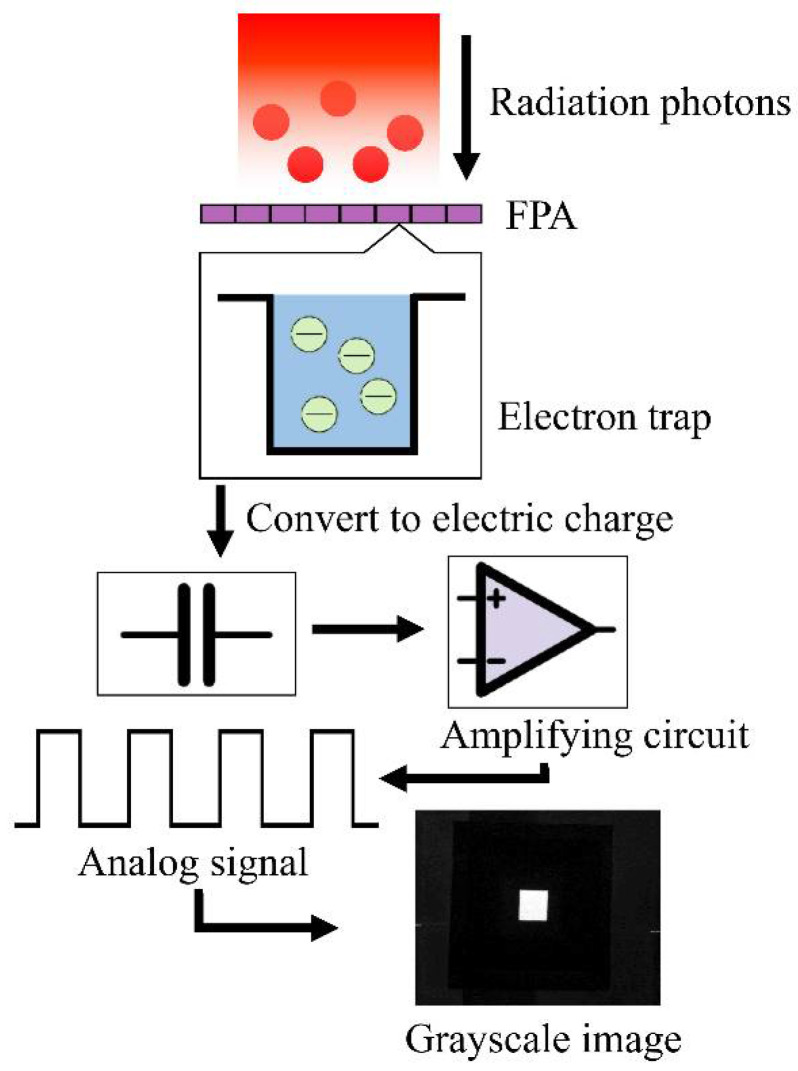
Physical model of the linear response process of the detector.

The linear response model of the cooled infrared focal plane array is the basis of infrared radiation characteristic measurement technology. The output digital image is a series of processes that transform the photoelectrons emitted from the infrared radiation source into the sensitive elements of the focal plane during a certain exposure time, thereby exciting a charge, and the accumulated charge generates a voltage, which is converted into a digital grayscale value [14,15]. This process is represented in Figure 1.

Assuming that the number of photons incident on the infrared measurement system is $N_{p}$ and the number of charges excited by photon radiation is $N_{e}$, the photoelectric conversion efficiency η(λ) of the detector is as follows:

(1) $$\eta \left(\lambda \right)=\frac{N_{p}}{N_{e}}$$

If the area of every pixel of the detector is $A_{p}$, the relationship between the radiant exitance M (in ${W/m}^{2}$) of the target and its photon number $N_{e}$ in a unit of time is as follows:

(2) $$M\left(\lambda ,T\right)=\frac{h\nu \cdot N_{p}}{A}=\frac{hc}{\lambda }\cdot \frac{N_{e}}{A\cdot \eta \left(\lambda \right)}$$

where c is the speed of light, which is $2.99\times 10^{8}\;m/s$, and h is Planck’s constant, which is $6.6260755\times 10^{-34}\;J\cdot s$.

For the infrared optical system with the detection band of $\lambda_{1}\sim \lambda_{2}$, if the size of the pixel is fixed, the accumulated charge on the focal plane of the pixel has a linear relationship with the emittance of the radiation source at a specific wavelength. The accumulated charge of the infrared FPA is converted into voltage, which is amplified by a linear circuit, and the linear magnification is recorded as K. Finally, the output digital gray value is DN. This process can be described as follows:

(3) $$DN=K\cdot N_{e}+B$$

where B is the internal offset of the detector, which is caused by the fact that the photosensitive material of the infrared focal plane itself and the subsequent optical system cannot be completely cooled to absolute temperature, and its radiation photons are also converted into digital grayscale.

### 2.2. Radiometric Calibration Model of the Near-Extended Area Blackbody Source

The infrared radiation characteristic measurement is based on the infrared image acquired by the system, and the radiation flux of the entrance pupil of the system is obtained; then, the radiation characteristic of the target is calculated. Therefore, it is necessary to obtain the response parameters of the infrared camera’s FPA through radiometric calibration, and to establish the quantitative relationship between the target radiation and the output gray value of the system [16,17].

**Figure 2:**
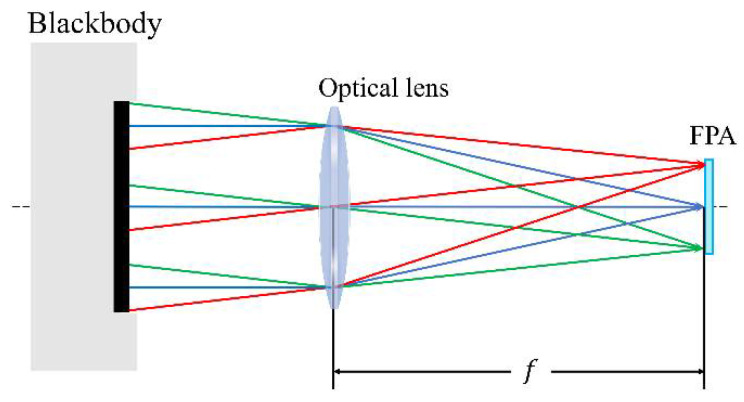
Schematic diagram of radiometric calibration using the near-extended area blackbody with geometric distribution of infrared radiation transmission.

The blackbody calibration method of near-extended source (NES) is the most common calibration method in the field of infrared radiometric measurement. An area blackbody source with high emissivity and good uniformity is used as the standard extended source to calibrate the system, and the effective radiation surface of the area blackbody source needs to completely cover the entrance pupil of the system. f is the focal distance of the optical lens. The calibration principle is shown in Figure 2.

According to Planck’s radiation law, the radiance emitted by a blackbody with emissivity of $\varepsilon_{bb}$ and temperature of $T_{bb}$ is as follows:

(4) $$L_{bb}\left(T_{bb}\right)=\varepsilon_{bb}L\left(T_{bb}\right)=\frac{\varepsilon_{bb}}{\pi }\int_{\lambda_{1}}^{\lambda_{2}}\frac{C_{1}}{\lambda^{5}\left(e^{C_{2}/\lambda T_{bb}}-1\right)}d\lambda$$

where $L(T_{bb})$ is the radiance emitted by an ideal blackbody, the first radiation constant is $C_{1}=\left(3.7415\pm 0.0003\right)\times 10^{8}\;W\cdot {\mu m}^{4}/m^{2}$, and the second radiation constant is $C_{2}=\left(1.43879\pm 0.00019\right)\times 10^{4}\;\mu m\cdot K$.

The radiant power $P_{bb}\left(T_{bb}\right)$ received by each image pixel from the calibrated blackbody can be expressed as follows [7,8]:

(5) $$P_{bb}\left(T_{bb}\right)=\tau_{opt}\left[\varepsilon_{bb}L\left(T_{bb}\right)\right]\frac{A_{p}}{f^{2}}\left[\pi {\left(\frac{D}{2}\right)}^{2}\right]=K_{p}\cdot L\left(T_{bb}\right)$$

For a given infrared system, $K_{p}=\frac{\pi \tau_{opt}\varepsilon_{bb}}{4}\cdot {\left(\frac{D}{f}\right)}^{2}\cdot A_{p}$ is a constant, $\tau_{opt}$ is the average spectral transmittance of the optical system in the corresponding wavelength band, D is the pupil diameter of the system, and f is the focal length of the system.

According to Equations (2) and (5), the calibration equation, which is the linear relationship between the output grayscale and the input radiance of the IR system, can be determined as follows:

(6) $$DN=R_{b}\cdot L\left(T_{bb}\right)+B_{in}$$

By changing the temperature of the blackbody, and through the fitting of temperature and response grayscale values, the response gain $R_{b}$ and response offset $B_{in}$ of the detector can be obtained.

### 2.3. Infrared System Calibration Model Considering Ambient Temperature and Stray Radiation

The radiation received by the detector also includes the spontaneous radiation of the infrared optical system and the reflected ambient radiation, which is closely related to the ambient temperature, and can be called stray radiation. When the environment changes, the output gray value shifts, which affects the calibration accuracy [18].

**Figure 3:**
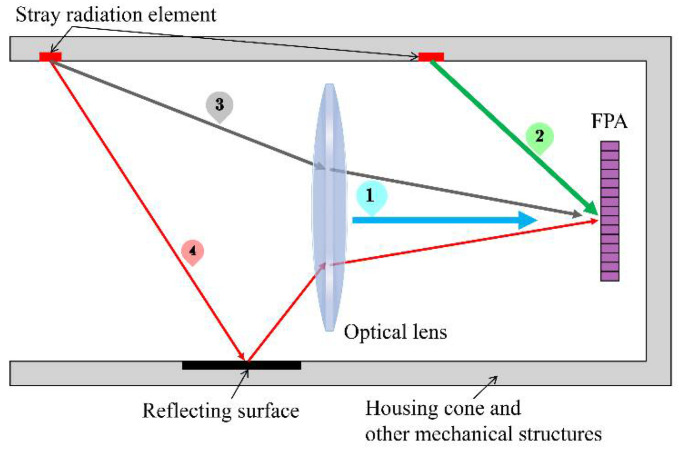
Geometry of stray radiation. ➀ self-radiation from the optical lens; ➁ radiation received directly by the detector from mechanical structures such as the housing cone; ➂ radiation from the housing cone and other mechanical structures through the lens to reach the detector; ➃ radiation from the housing cone and other mechanical structures after reflection through the lens into the detector.

For infrared imaging systems, stray radiation comes mainly from the radiation of the lens, the housing cone, and other mechanical structures. The radiation of the lens and other components is determined by their own temperature. Due to their thermal conductivity, the temperature of the components becomes uniform. In this paper, all components except for the detector are assumed to be at an ambient temperature [19,20]. Figure 3 shows a schematic diagram of the stray radiation transmission of the imaging system.

The stray radiation received by the detector consists of four main parts: (1) self-radiation from the optical lens; (2) radiation received directly by the detector from mechanical structures such as the housing cone; (3) radiation from the housing cone and other mechanical structures through the lens to reach the detector; and (4) radiation from the housing cone and other mechanical structures after reflection through the lens into the detector.

In summary, when considering the ambient temperature and stray radiation, the radiant power received by the detector at the time of calibration can be expressed as follows:

(7) $$P_{cl}=P_{bb}\left(T_{bb}\right)+P_{l}\left(T_{amb}\right)+P_{s}\left(T_{amb}\right)+P_{nar}$$

where the narcissus radiation power $P_{nar}$ is the radiation of the cooled detector reflected by the optics, which can be weakened by reducing the reflectivity of the core surface, and is independent of the ambient temperature [16].

$P_{l}\left(T_{amb}\right)$ represents the spontaneous radiation of the optical lens:

(8) $$P_{l}\left(T_{amb}\right)=L_{l}\left(T_{amb}\right)A_{p}\Omega_{l}$$

where $L_{l}$ is the radiant brightness of the lens at ambient temperature $T_{amb}$, which is related to the emissivity of the lens; $\Omega_{l}$ is the projected solid angle of the system’s pupil as seen from the pixel; and $A_{p}$ is area of the pixel.

$P_{s}(T_{amb})$ is the stray radiation generated by the housing cone and the other mechanical structures:

(9) $$P_{s}\left(T_{amb}\right)=K_{i}L\left(T_{amb}\right)+K_{j}L\left(T_{amb}\right)+K_{k}L\left(T_{amb}\right)$$

where $K_{i}=\sum_{i}^{n}\varepsilon \left(\theta_{i},\varphi_{i}\right)A_{i}\Omega_{i}$, $K_{j}=\sum_{j}^{m}\varepsilon \left(\theta_{j},\varphi_{j}\right)A_{j}\Omega_{j}\tau_{l}$, $K_{k}=\sum_{k}^{q}\varepsilon \left(\theta_{k},\varphi_{k}\right)A_{k}\Omega_{k}\rho_{s}\tau_{l}$, $\varepsilon \left(\theta ,\varphi \right)$ is the emissivity of the stray radiation element, its area is A, $\Omega_{i,j,k}$ denotes the projected solid angle, $\tau_{l}$ is the transmittance of the optical lens, and $\rho_{s}$ is the path reflectance. $K_{i,j,k}$ is the theoretical constant for a given infrared system, and the flux resulting from stray radiation of the system is directly proportional to the radiance of an ideal blackbody at ambient temperature.

Combining Equations (5)–(9), we can obtain the infrared system calibration model considering the ambient temperature:

(10) $$DN=R_{b}\cdot L\left(T_{bb}\right)+R_{s}\cdot L\left(T_{amb}\right)+B_{in}$$

where $R_{s}$ is the response gain of the stray radiation related to ambient temperature.

## 3. Equivalent Calibration of Infrared Systems Based on Blackbody Baffle

### 3.1. Establishment of the Equivalent Calibration Conversion Function Model

**Figure 4:**
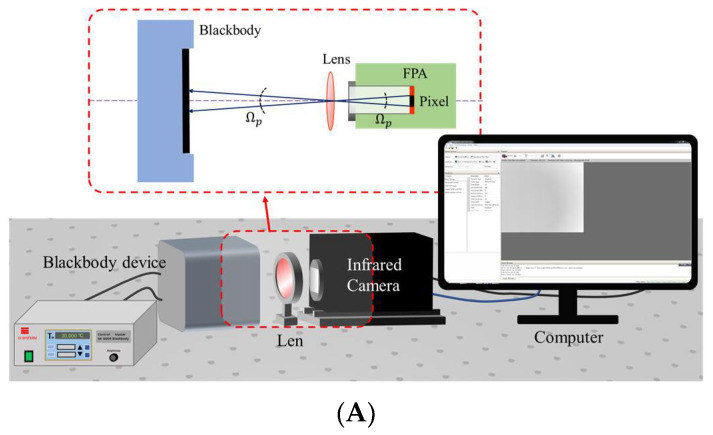

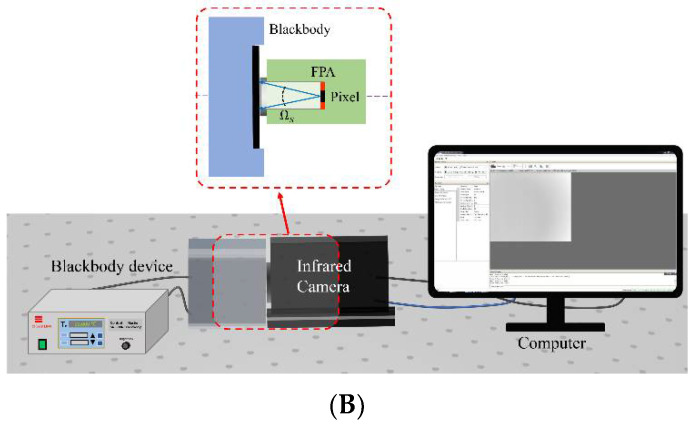
(**A**) Schematic diagram of the device for the calibration of the whole infrared system; geometric optical distribution of the infrared radiation at the detector pixel when observing the external blackbody. (**B**) Schematic of detector calibration based on blackbody baffle; geometric optical distribution of infrared radiation at the pixel.

At present, the system calibration of large aperture-infrared radiation characteristic measurement equipment relies on the large-area blackbody source matching its aperture, which brings higher costs and a greater workload to the calibration work, making it comparatively cumbersome to carry out. To solve the above problems, an equivalent calibration method for IR systems based on blackbody baffle is proposed. The method needs to fit the correspondence between the infrared image system calibration and the blackbody baffle calibration, and its working principle is shown in Figure 4. Figure 4A shows the schematic diagram of the calibration of the whole infrared system, while Figure 4B shows the schematic diagram of the detector calibration based on blackbody baffle.

According to the derivation of the equations in Section 2, the radiant power $P_{cl}$ received at the pixel of the whole infrared system calibration is as follows:

(11) $$P_{cl}=L_{bb}\left(T_{bb}\right)A_{l}\Omega_{p}\tau_{l}\tau_{atm}+L_{l}\left(T_{amb}\right)A_{p}\Omega_{l}+\left(K_{i}+K_{j}+K_{k}\right)L\left(T_{amb}\right)+P_{ref}+P_{nar}$$

where $P_{ref}$ is the reflected radiant power of the background environment from the lens.

When using the blackbody baffle for direct calibration, the radiant power $P_{cs}$ received by the pixel is as follows:

(12) $$P_{cs}=L_{s}\left(T_{s}\right)A_{p}\Omega_{s}$$

Equation (11) describes the relevant physical quantities in Figure 4A, where $L_{bb}$ is the radiance emitted by the external blackbody at temperature $T_{bb}$; $A_{p}$ is the area of the optical system pupil (mid-wave infrared lens); $\Omega_{p}$ is the projected solid angle of the pixel’s instantaneous field of view, determined by the pixel area $A_{p}$ and the distance from the detector to the pupil of the system; $\tau_{l}$ is the spectral transmittance of the infrared lens; $\tau_{atm}$ is the spectral transmittance of the atmosphere, which is approximately equal to 1 at a short distance and in the indoor environment; $L_{l}$ is the radiance of the lens at ambient temperature $T_{amb}$; $\Omega_{l}$ is the projected solid angle of the lens as seen from the pixel; and $K_{i,j,k}$ represents the coefficients related to stray radiation.

In Equation (12), $L_{s}$ is the radiance emitted by the blackbody baffle as a function of its temperature $T_{s}$, and $\Omega_{s}$ is the projected solid angle when viewing the baffle from the pixel, determined by the size of the cold aperture of the infrared camera and the distance between the detector and the cold aperture.

In order to characterize the relationship between the two calibration methods, we define an equivalent calibration conversion function $E_{c}$, as follows:

(13) $$T_{bb}=T_{s}=T_{1}E_{c}\left(T_{1}\right)=\frac{DN_{cl1}-B_{in}}{DN_{cs1}-B_{in}}$$

(14) $$E_{c}=\frac{P_{cl}}{P_{cs}}$$

where the emissivity of the lens is $\varepsilon_{l}$ and the reflectivity is $\rho_{l}$ at ambient temperature $T_{amb}$, and the radiance of the lens is equal to the radiance emitted by an ideal blackbody with emissivity of $\varepsilon_{l}$:

(15) $$L_{l}\left(T_{amb}\right)=\varepsilon_{l}L\left(T_{amb}\right)$$

(16) $$P_{ref}=\rho_{l}L\left(T_{amb}\right)$$

(17) $$P_{nar}=\rho_{l}\varepsilon_{fpa}L\left(T_{fpa}\right)$$

In cooled infrared radiation measurement systems, the detector is in a low-temperature environment, and the value of $P_{nar}$ is small enough, so we ignore the narcissus in this article for the sake of simplicity.

When the external blackbody is used as the baffle blackbody, and both have the same temperature setting, there is:

(18) $$L_{s}\left(T_{s}\right)=\varepsilon_{bb}L\left(T_{bb}\right)$$

In summary, the equivalent calibration conversion function at a certain ambient temperature can be simplified as follows:

(19) $$E_{c}\left(T_{bb}\right)=\frac{A_{l}\Omega_{p}\tau_{l}}{A_{p}\Omega_{s}}+\frac{\left[\varepsilon_{l}A_{p}\Omega_{l}+\left(K_{i}+K_{j}+K_{k}\right)+\rho_{l}\right]L\left(T_{amb}\right)}{A_{p}\Omega_{s}}\cdot \frac{1}{L\left(T_{bb}\right)}$$

At a certain ambient temperature, there is only one variable in Equation (18) for a certain infrared radiation characteristic measurement system; that is, $E_{c}$ is a function of the blackbody temperature.

### 3.2. Measurement of the Equivalent Calibration Conversion Function

The output grayscale of the infrared camera is proportional to the radiant power received by the detector, so the relationship between the grayscale response DN of the camera and $E_{c}$ in both cases can be written as follows:

(20) $$DN_{cl}-B_{in}=\left(DN_{cs}-B_{in}\right)E_{c}\left(T_{bb}\right)$$

where $DN_{cl}$ is the grayscale response of the optical system when observing the external blackbody, $DN_{cs}$ is the grayscale response of the detector when directly observing the baffle blackbody, and $B_{in}$ is the grayscale value generated by the internal offset of the detector—independent of the input radiant power. Through the equivalent calibration conversion function, the response of the baffle blackbody can be converted to the equivalent external blackbody response to complete the calibration conversion.

The specific measurement process of the function for the baffle blackbody’s temperature-dependent ratio $E_{c}(T_{bb})$ is as follows: A series of blackbody temperatures are set in the camera’s temperature measurement range with the same indoor environment. By alternately measuring a series of optical imaging system calibration images and baffle blackbody calibration images at the same temperature, the ratio of their gray values can be calculated, as follows:

(21) $$\begin{array}{l} T_{bb}=T_{s}=T_{1},E_{c}\left(T_{1}\right)=\frac{DN_{cl1}-B_{in}}{DN_{cs1}-B_{in}}\\ T_{bb}=T_{s}=T_{2},E_{c}\left(T_{2}\right)=\frac{DN_{cl2}-B_{in}}{DN_{cs2}-B_{in}}\\ T_{bb}=T_{s}=T_{3},E_{c}\left(T_{3}\right)=\frac{DN_{cl3}-B_{in}}{DN_{cs3}-B_{in}}.\\ \ldots \;\;\;\;\;\ldots \\ T_{bb}=T_{s}=T_{i},E_{c}\left(T_{i}\right)=\frac{DN_{cli}-B_{in}}{DN_{csi}-B_{in}} \end{array}$$

Because there is almost no stray radiation in the calibration of the baffle blackbody, the internal offset $B_{in}$ of the detector can be calculated by the calibration fitting curve of the baffle blackbody.

After enough data are collected, the mathematical relationship between the equivalent calibration conversion function $E_{c}$ and the blackbody temperature $T_{bb}$ can be determined by numerical fitting, and the gray response of the equivalent external blackbody at any temperature can be calculated using this relationship.

## 4. Laboratory Measurements

In order to verify the theories elaborated above, the experiments were conducted in two parts, in a laboratory with a relatively stable environment. Firstly, radiation calibration experiments were performed in the laboratory to determine the equivalent conversion function. In the second part, imaging experiments were performed to acquire infrared images of the target blackbody at different temperatures, and the radiometric radiance inversion of the images was performed using the equivalent calibration conversion function measured in the first part, and compared with the actual values.

A cooled mid-wave infrared (MWIR) camera with a forward-looking infrared (FLIR) system and a large-scale mercury cadmium telluride (MCT) focal plane array (FPA) with 320×256 pixels was selected for the radiometric calibration experiments. The experiments were conducted using a mid-wave infrared lens with a focal length of 50 mm, an aperture of 25 mm, and a transmittance of about 0.9. An area blackbody source with highly effective emissivity was selected as the calibrated blackbody. The specific parameters of the camera and the blackbody device are given in Table 1 and Table 2, respectively.

### 4.1. Experimental Measurement of the Equivalent Calibration Conversion Function

**Figure 5:**
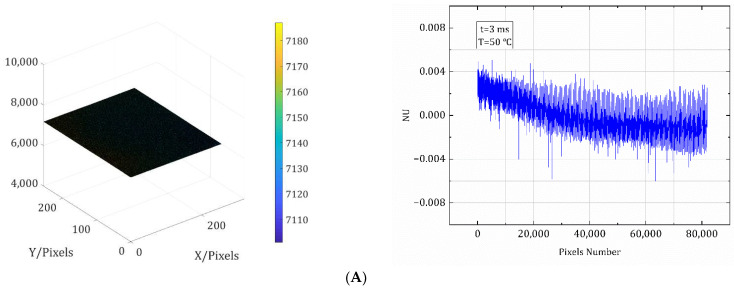

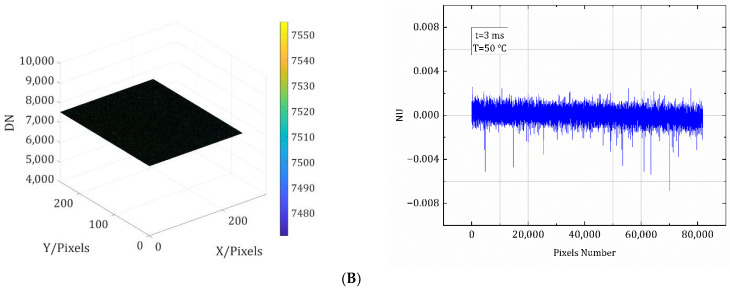
(**A**) Grayscale distribution of the detector’s target surface at 3 ms and 50 °C, and non-uniformity of each pixel of the correction image, with lens. (**B**) Grayscale distribution of the detector’s target surface at 3 ms and 50 °C, and non-uniformity of each pixel of the correction image, without lens.

Non-uniformity correction of the detector is required prior to IR calibration. Although the thermal radiation from the blackbody radiation source is uniform, the response of the detector is indeed inhomogeneous due to reasons such as manufacturing, which requires non-uniformity correction to achieve its uniform response [21]. Since this article focuses on the calibration method, it does not provide the non-uniformity correction process. All calibration images in this paper were corrected using a non-uniformity correction algorithm. The grayscale distribution of the calibrated images and the non-uniformity of their pixels at a certain temperature at a certain integration time are given in Figure 5 as an example to show that the detector is uniformly illuminated. In both cases, the non-uniformity of the pixels is within 0.8%, indicating a uniform detector response.

**Figure 6:**
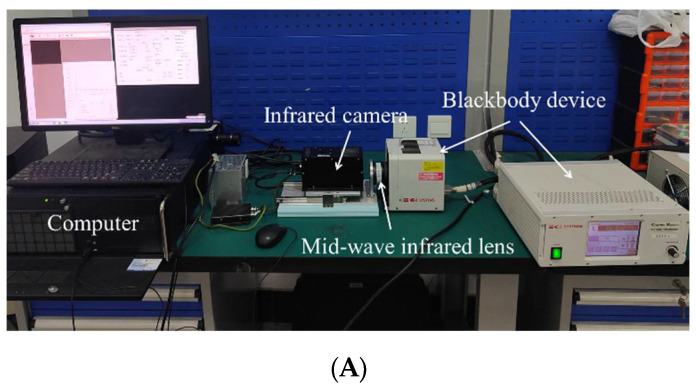

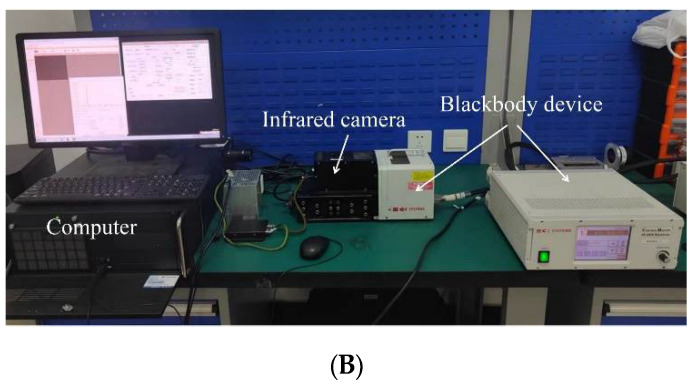
(**A**) Experimental setup for the calibration of the near-extended source (NES) blackbody. (**B**) Calibration experiment of the simulated baffle blackbody.

In a stable laboratory environment, the calibration experiment was set up as shown in Figure 6. The blackbody temperature was ramped up from 25 °C to 70 °C at 5 °C intervals, and the gray images corresponding to different integration times at each temperature were acquired. The same blackbody was used throughout the experiment.

**Figure 7:**
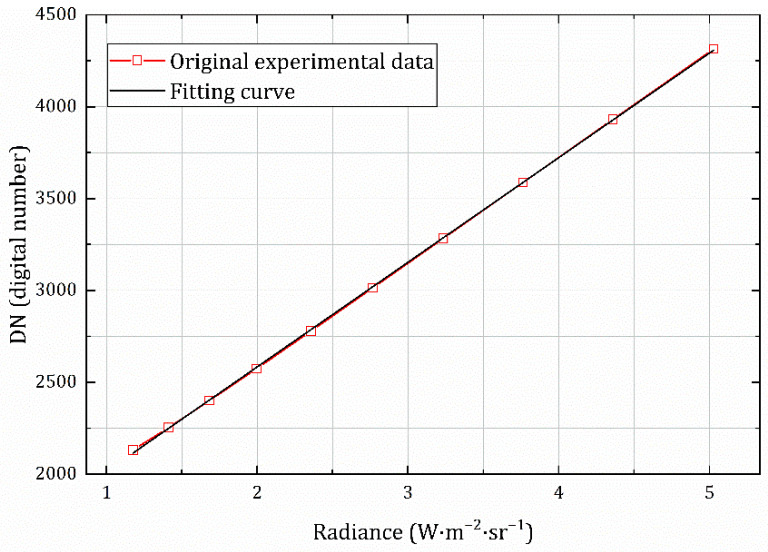
Baffle blackbody calibration curve with integration time of 1 ms.

The radiance value of the corresponding temperature of the baffle blackbody was fitted with the acquired image grayscale value by the least squares method to obtain the calibration equation (1 ms integration time) given by Equation (12), and the value of $B_{in}$ was calculated as 1445.80702 (digital number). The fitting curve is shown in Figure 7, and the goodness of fit of the calibration curve is 0.99987; this is also called the coefficient of determination, which represents the degree of fit of the regression line to the observed values. The closer the value is to 1, the higher the degree of explanation of the dependent variable by the independent variable.

(22) $$DN_{cs}=569.31976\cdot L\left(T_{bb}\right)+1445.80702$$

Using Equation (21), the acquired grayscale images in the experiments were data-processed to determine the values of the equivalent calibration conversion function at the corresponding radiance, which are given in Table 3. Based on the calculated results, the mathematical expression of the conversion function was obtained by fitting the radiance to the corresponding value of $E_{c}$:

(23) $$E_{c}\left(T_{bb}\right)=0.897+0.11046/L\left(T_{bb}\right)$$

**Figure 8:**
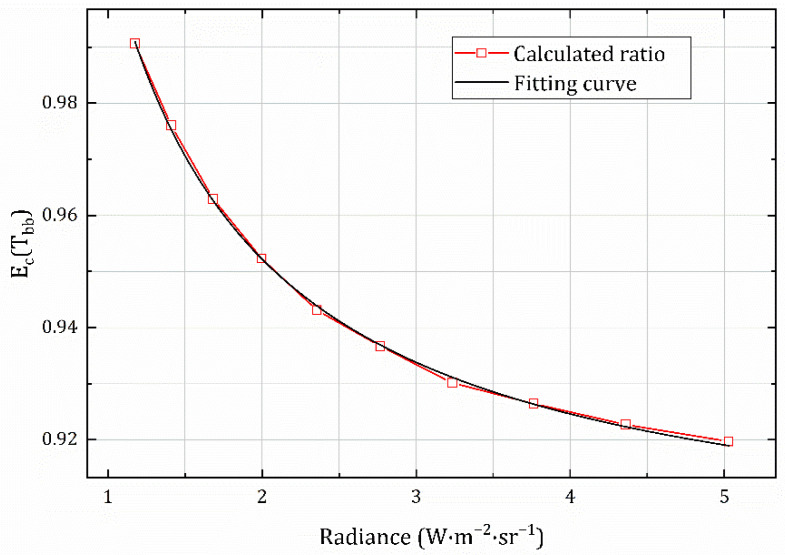
Fitting curve of the equivalent calibration conversion function.

The fitting curve is shown in Figure 8, and the goodness of fit is 0.99931. The fitting Equation (22) is consistent with Equation (18), indicating that the data are valid and that the theoretical derivation process is correct and reasonable.

### 4.2. Imaging Experiments and the Inversion Data

**Figure 9:**
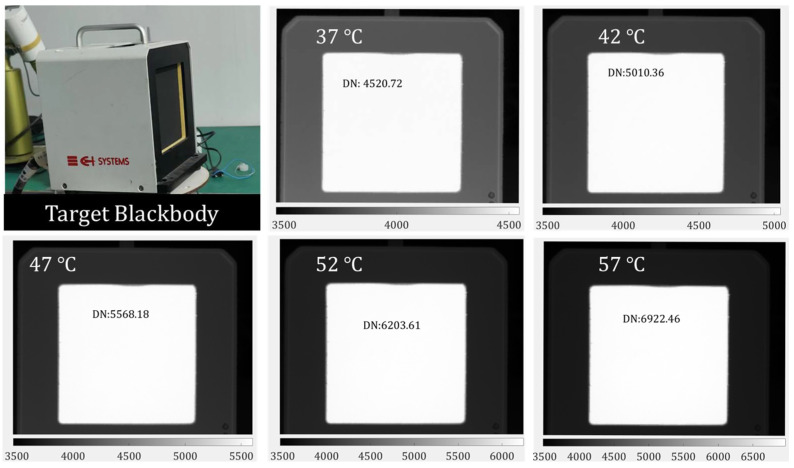
Target blackbody and its imaging at different temperatures.

To prove the correctness of the equivalent calibration conversion function (ECCF) of Equation (22), target blackbody images at different temperatures (Figure 9) were acquired, and the inversion of the radiance was performed according to the equivalent calibration of Equation (23), determined by the fitted Equations (19) and (22).

Meanwhile, as with Equation (22), the calibration of Equation (24) for the external near-extended source (NES) method was determined by fitting the experimental data; the inversion of the radiance of the target image was also performed, and the calibration of Equation (25) of the ECCF method was determined according to Equation (23). The inversion results were analyzed in comparison with the actual radiance corresponding to the blackbody temperature, using two methods.

**Figure 10:**
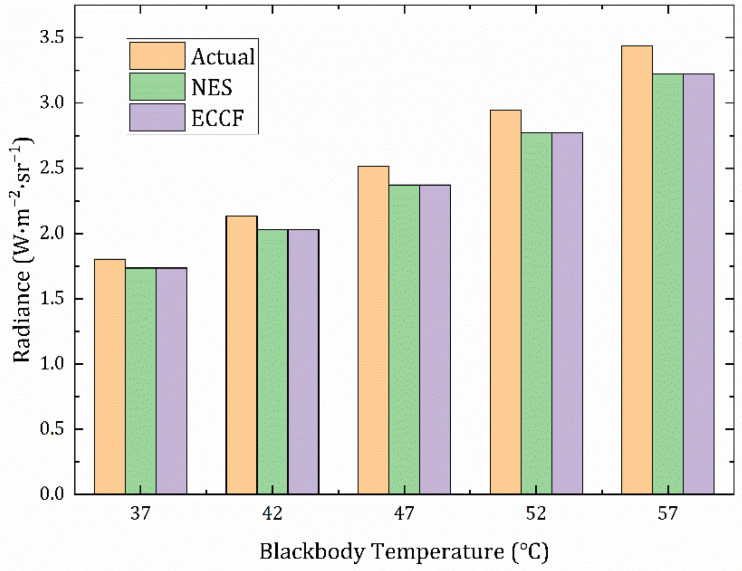
Comparison of the inversion radiance of the two methods with the actual radiance of the target.

**Figure 11:**
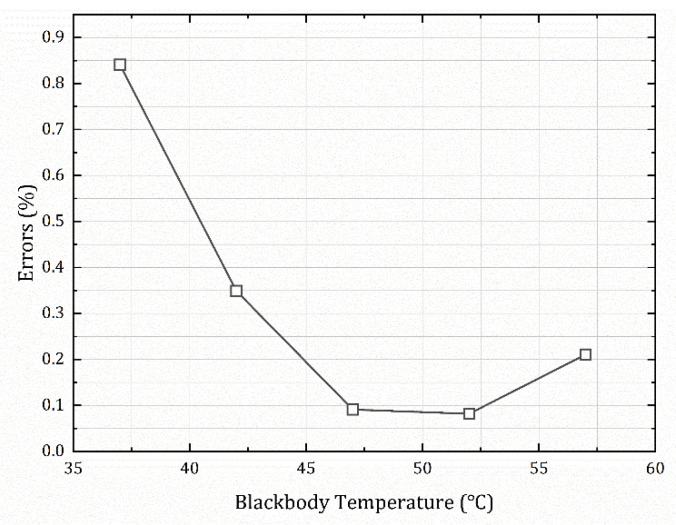
The errors (1 ms) between the processed data of the two methods.

The data are listed in Table 4, and Figure 10 shows a visual comparison of the radiance calculated by the two methods with the actual radiance values of the target. Figure 11 shows the error between the data processed by the external blackbody’s direct calibration method and the data processed by the method proposed in this paper at different temperatures.

(24) $$DN_{ce}=510.56214\cdot L\left(T_{bb}\right)+1509.08517$$

(25) $$DN_{cl}=510.91381\cdot L\left(T_{bb}\right)+1508.18517$$

Equations (24) and (25) are essentially the same. Based on the inversion results in Figure 10 and the inversion errors in Figure 11, they are also consistent; the average error between the two methods is only 0.198%, and the maximum error does not exceed 1%. This shows that it is reasonable to derive an equivalent calibration conversion function for the measurements that can be applied. We calculated the error between the measurement results of the two methods and the actual value, and the maximum did not exceed 7%. The inversion accuracy fully met the requirements of the infrared radiant measurement equipment work, as shown in Table 5. According to the related literature [22,23], the accuracy of infrared radiation characteristic measurement of the target is generally about 15% at present, so 7% is completely acceptable, and the accuracy is relatively high.

In addition, we used the constant scale equivalent conversion function E, calculated at an integration time of 1 ms, to process grayscale images at different integration times; the errors between the calculated results and the actual radiation of the target are listed in Table 6. The maximum error was within 6%.

**Figure 12:**
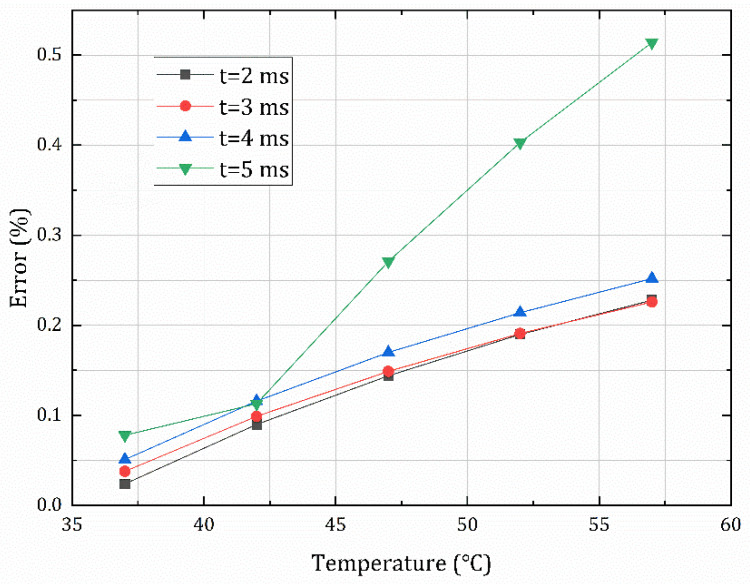
The error distribution between the ECCF method and the NES method at different integration times.

The error distribution between the two methods at different integration times and target temperatures is shown in Table 7 and Figure 12. As can be seen from the figure, the error distribution is independent of the integration time, and all of the errors are within 0.6%, indicating that $E_{c}(T_{bb})$ measured at a certain integration time can be generalized to the conversion of the calibration equation for other integration times.

**Figure 13:**
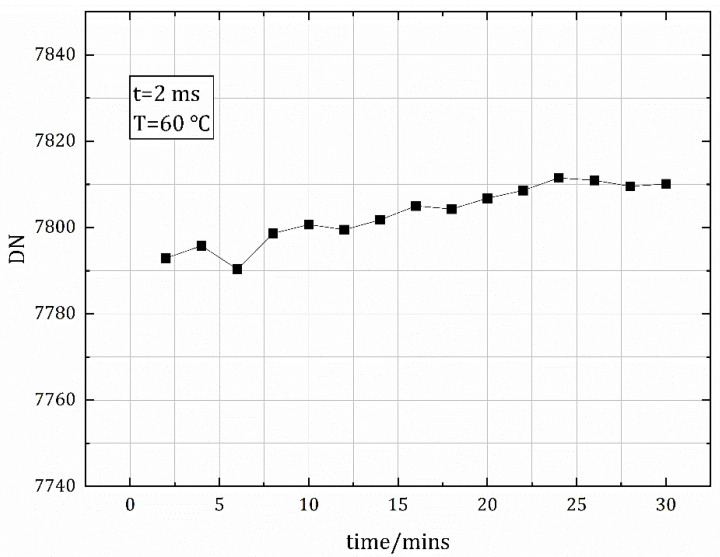
Image grayscale values over time when the blackbody is set to 60 °C (integration time = 2 ms).

Finally, we added an experiment. The blackbody baffle was placed in front of the camera for image acquisition, and the blackbody temperature was set to 60 °C. A calibration image was acquired every two minutes for 30 min. The results are shown in Figure 13. It can be seen that the image’s gray value does not change much with time—within 0.3%—indicating that the blackbody heat source has little effect on the temperature change of the internal components of the cooled camera, and the detector response is effectively unchanged. This further illustrates the feasibility of the calibration method for application to radiometric characteristic measurement equipment using a cooled infrared camera.

## 5. Conclusions

This paper presents an idea of replacing the large surface-source blackbody calibration for fieldwork. This is an equivalent calibration conversion method based on an internal blackbody baffle. Based on the calibration model considering the ambient temperature, the specific form of the $E_{c}(T_{bb})$ (equivalent calibration conversion function (ECCF)) was derived. The calibration experiments were performed using a cooled mid-wave infrared camera, the ECCF was measured and calculated, and the proposed method was verified and evaluated by target imaging experiments. The results show that the calibration results of the system achieved by the conversion function are generally consistent with the direct calibration results of the external blackbody, and the error between them is within 1% (1 ms). The error of the inversion results using this method also meets the measurement accuracy with the actual radiance. Therefore, when the equipment is working in the external field, only a small blackbody baffle is needed to calibrate the detector, and using the conversion function, the equivalent results of direct calibration of a large surface-source blackbody covering the aperture of the equipment can be obtained, reducing the workload, working time, and equipment maintenance costs of equipment calibration.

## Figures and Tables

**Table 1:** Parameters of the cooled infrared camera.

Materials	HgCdTe
Spectral range	3.7 μm ~4.8 μm
Aperture	f/4
Pixel size	15 µm × 15 μm
Digital output depth	14
Resolution	320 (H) × 256 (V)
Operating temperature	−40 °C~+60 °C

**Table 2:** Parameters of the area source blackbody.

Blackbody emitter size	100 mm × 100 mm
Operating temperature range	0 °C~125 °C
Temperature accuracy	0.01 °C
Effective emissivity	0.97
Operating temperature head	−20 °C~70 °C
Operating temperature controller	0 °C~50 °C

**Table 3:** Experimental data and calculation of $E_{c}(T_{bb})$.

Tem./°C	Radiance/$W\cdot m^{-2}\cdot {\mathrm{sr}}^{-1}$	$DN_{cl}$/Digital Number	$DN_{cs}$/Digital Number	$E_{c}(T_{bb})$
25	1.17567	2125.09	2131.52	0.99063
30	1.41061	2234.29	2253.64	0.97605
35	1.68279	2364.90	2400.25	0.96296
40	1.99649	2520.64	2574.43	0.95234
45	2.35631	2702.67	2778.50	0.94310
50	2.76712	2914.86	3014.11	0.93672
55	3.23408	3155.04	3283.44	0.93013
60	3.76264	3430.11	3587.63	0.92646
65	4.35851	3738.66	3930.68	0.92272
70	5.02770	4084.60	4314.93	0.91972

**Table 4:** The inversion data of two calibration methods, and the error between them.

Tem./°C	Radiance/$W\cdot m^{-2}\cdot {\mathrm{sr}}^{-1}$			Er./%
	Actual	NES	ECCF	
37	1.80303	1.73559	1.73502	0.841
42	2.13462	2.03034	2.02998	0.349
47	2.51424	2.37207	2.37194	0.091
52	2.94687	2.76978	2.76992	0.082
57	3.43780	3.22122	3.22168	0.211

**Table 5:** The error of calculation results of two methods and the actual value.

Tem./°C	NES Error/%	ECCF Error/%
37	3.740	3.772
42	4.885	4.902
47	5.654	5.660
52	6.009	6.005
57	6.300	6.287

**Table 6:** Inversion errors of different integration times between the ECCF method and the actual value.

Tem./°C	Error of Different Integration Times/%			
	2 ms	3 ms	4 ms	5 ms
37	3.607	3.635	3.580	3.695
42	4.194	4.195	4.168	4.177
47	4.763	4.794	4.719	4.621
52	5.213	5.260	5.214	5.047
57	5.649	5.663	5.585	5.370

**Table 7:** The errors of different integration times between the ECCF method and the NES method.

Tem./°C	Error of Different Integration Times/%			
	2 ms	3 ms	4 ms	5 ms
37	0.024	0.038	0.051	0.078
42	0.090	0.099	0.116	0.113
47	0.144	0.149	0.170	0.271
52	0.190	0.191	0.214	0.403
57	0.228	0.226	0.252	0.514

## Data Availability

The data presented in this study are available upon request from the corresponding author.
